# Impact of social inequalities at birth on the longevity of children born 1914–1916: A cohort study

**DOI:** 10.1371/journal.pone.0185848

**Published:** 2017-10-16

**Authors:** Nicolas Todd, Sophie Le Fur, Pierre Bougnères, Alain-Jacques Valleron

**Affiliations:** 1 INSERM U1169, Le Kremlin-Bicêtre, France; 2 Médecine des Adolescents, Hôpital Bicêtre, Paris Sud University, Le Kremlin-Bicêtre, France; Centre Hospitalier Universitaire Vaudois, FRANCE

## Abstract

**Background:**

Testing whether familial socioeconomic status (SES) in childhood is a predictor of mortality has rarely been done on historical cohorts.

**Methods:**

The birth certificates of 4,805 individuals born 1914–1916 in 16 districts of the Paris region were retrieved. The handwritten information provided the occupation of parents, the legitimacy status, life events (e.g. marriage, divorce), and the precise date of death when after 1945 (i.e. age 31 years (y) in the cohort). We used the median age at death (MAD) as a global measure of mortality, then studied separately survival to and after 31 y. Multivariate Imputation by Chained Equations (MICE), Generalized Additive Models (GAMs) and mixed effect Cox models were used.

**Results:**

MAD showed large variations according to paternal occupation. The lowest MAD in both sexes was that of workers’ children: it was 56.3 y (95% CI: [48.6–62.7]) in men and 67.4 y (95% CI: [60.8–72.7]) in women, respectively (95% CI: 13.4 y [5.7–21.3]) and 12.3 y (95% CI: [4.0–19.2]) below the highest MAD attained. MAD experienced by illegitimate children was 18.9 y (95% CI: [13.3–32.3]) shorter than of legitimate children. The multivariate analysis revealed that in both sexes survival to age 31 y was predicted independently by legitimacy and paternal occupation. Paternal occupation was found significantly associated with mortality after age 31 y in females only: accordingly difference in life expectancy at age 31 y was 4.4 y (95% CI: [1.2–7.6]) between upper class and workers’ daughters.

**Conclusions:**

Paternal occupation and legitimacy status were strong predictors of offspring longevity in this one-century historical cohort born during World War One.

## Introduction

The French civil registration system is organized so that all major life events (such as marriages, divorces and deaths) are systematically notified on birth certificates, wherever these events occur. This has previously provided us with the opportunity to study the adult mortality of orphans who lost their father during World War One (WW1) [1]. The same characteristic of the French civil registration system also offers a rare opportunity for the study of whole-life mortality according to socioeconomic status (SES) at birth.

The effect of SES in early life on health across the lifespan has recently raised a growing interest among epidemiologists [2–4]. Parental SES influences nutrition, maternal stress, and exposure to infections, and can therefore act on physical parameters of a child’s development during specific windows of plasticity, notably in intra-uterine life and infancy [5, 6]. Parental SES may also act on future health because it influences educational attainment and thus contributes to SES reached in adulthood, itself a strong predictor of mortality [7]. In addition, wealth is usually transmitted from parents to offspring and may affect health directly or through access to healthcare.

A global assessment of the effect of parental SES on mortality requires the study of quasi-extinct cohorts. Obviously, data on such cohorts are very difficult to obtain, so that most studies about childhood SES have examined adult mortality on short age spans, typically below 30 years. One notable exception is the recent analysis by Juárez et al. of a Swedish cohort born 1915–1929 in Uppsala, whose analysis was restricted to individuals for which at least one of the two parental occupations was known, and was stratified by age. Low SES at the time of the birth, defined by parental occupation (paternal occupation if available, maternal occupation otherwise), and marital status (i.e. legitimacy of the birth), was associated with an increase in mortality at all ages [8]. We report here an analysis of whole-life mortality according to both parental occupations at the time of birth and the legitimacy status of the birth for a cohort born 1914–1916 in Paris.

## Materials and methods

### Historical material used

#### Extraction of information from birth registers

**Fig 1:**
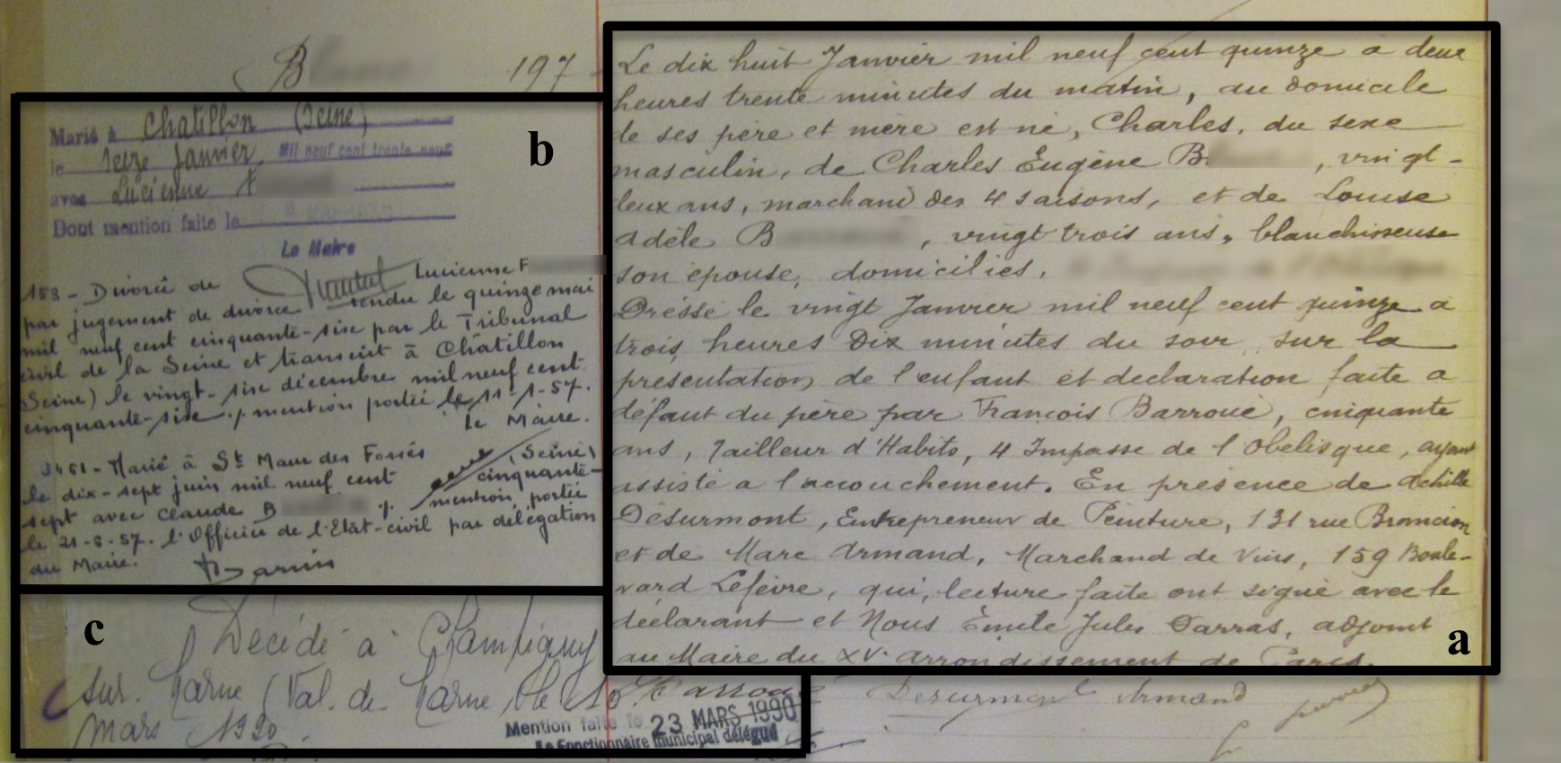
1914 French birth certificate. The birth certificate gives the date of birth, sex and parental occupations at the time of the birth (zone a). The date of death, if after March 29^th^ 1945, is available in the margin of the birth certificate (zone c). The dates of other life events (zone b) such as marriages and divorces are also notified by law in the margin (since 1897 for marriages, 1939 for divorces) and are used to classify those without a date of death. When no life event is notified after March 29^th^ 1945, the individual is considered dead before this date. Conversely, individuals without a date of death but known to be alive on March 29^th^ 1945 because of a life event after this date are considered alive at age 99. The birth certificate shown was registered on January 20^th^ 1915 and is held by the city hall of the 15^th^ arrondissement of Paris.

The data that we analyzed here are those of the 4,805 “control” subjects of a project on the health consequences of WW1. They are matched for date of birth, sex and age of the mother to the “pupilles de la Nation” (orphans or children of soldiers severely disabled during WW1) born between August 1^st^ 1914 and December 31^st^ 1916 in 16 administrative districts of the Paris region (14 “arrondissements” (boroughs) of Paris and two suburban cities, Neuilly-sur-Seine and Le Kremlin-Bicêtre). Though they were matched to the “pupilles de la Nation”, we anticipate the results obtained on this cohort may be generalized to the population of all Parisian births of the time. The following pieces of information were collected on the handwritten birth registers in each district’s city hall: date of birth, sex, parental age (precision: year) and occupation (see below) at the time of the birth, legitimacy status, date of death and date of the other life events notified in the margin of birth certificates (Fig 1).

The National Commission on Information Technology and Liberties (Commission Nationale de l’Informatique et des Libertés [9]) granted us authorization to access and analyse those data (registration number 915774).

#### Longevity information available on birth registers

By law, since March 29^th^ 1945, at occurrence of death, the civil registration service at the place of death systematically notifies the civil registration service at the place of birth, so that the birth certificate is updated in a few weeks at most. For individuals who died abroad, information is transmitted to the place of birth by the local French consulate. Given that data collection took several months, all dates of death were checked at the end (Sept. 29 –Oct. 8 2015) to avoid differences in the time period covered between districts due to different data collection dates. Because observation of death was available from 1945 onward, there was a 29-month difference in the *age* period on which individuals born in early August 1914 and those born at the end of 1916 were observed. [31; 99] years is the age range on which all dates of death are known.

An individual without a date of death on his birth certificate may have died before March 29^th^ 1945 or still be alive. An individual who was never observed after March 29^th^ 1945 (i.e. for whom no notification of marriage, divorce or guardianship is present on the birth certificate after this date) was considered dead before age 31 y. In the analyses of life expectancy at 31 y (see Statistical analysis below), the longevity L_31_ of those alive at age 99 y (~ 2.5% of all individuals included) was set to 99 + e_r_, where e_r_ is the life expectancy remaining at age 99 as given by the Human Mortality Database [10] for the French 1915 cohort (2.10 y for men and 2.52 y for women).

#### Classification of occupations

We defined 8 categories for maternal occupations at birth: worker, servant, craftswoman, employee, shopkeeper, housekeeper, housewife, middle & upper class. Housewife (“sans profession”) and housekeeper (“ménagère”) were occupations so frequently found on birth certificates that we made them categories in our classification of occupations. We defined 6 categories for paternal occupations at birth: worker, craftsman, employee, shopkeeper, middle class and upper class (Table 1). The classification of occupations was performed with all other information made unavailable, so that the investigator classifying (NT) was blind regarding the longevity associated with each occupation. The maternal occupation at the time of birth was available on 99.6% of birth certificates (n = 4,785). Except for oversights, the paternal occupation was available for legitimate children, but not for illegitimate children. We failed to classify 0.3% of all maternal occupations (n = 14) and 0.2% of all paternal occupations (n = 8). To impute the unavailable and unclassified paternal and maternal occupations, we performed multivariate imputation by chained equations (MICE) [11] using all variables in model (1) (see below) as predictors. We thereby created 10 completed datasets, on which all regression analyses were conducted. The regression coefficients obtained on the 10 datasets were then combined by Rubin’s rules [12, 13].

**Table 1:** Baseline characteristics of subjects.

	Legitimate births	Illegitimate births	All births
	(n = 3,424)	(n = 1,381)	(n = 4,805)
**Date of birth, mean**	1915-06-17	1915-06-27	1915-06-20
**Sex, n (%)**			
Female	1,637 (47.8%)	681 (49.3%)	2,318 (48.2%)
Male	1,787 (52.2%)	700 (50.7%)	2,487 (51.8%)
**Paternal occupation, n (%)**			
Worker	993 (29.0%)	299 (21.7%)	1,292 (26.9%)
Craftsman	593 (17.3%)	508 (36.8%)	1,101 (22.9%)
Employee	985 (28.8%)	277 (20.0%)	1,262 (26.3%)
Shopkeeper	312 (9.1%)	138 (10.0%)	450 (9.4%)
Middle class	198 (5.8%)	89 (6.4%)	287 (6.0%)
Upper class	342 (10.0%)	71 (5.1%)	412 (8.6%)
**Maternal occupation, n (%)**			
Worker	364 (10.6%)	201 (14.6%)	566 (11.8%)
Servant	241 (7.0%)	426 (30.8%)	667 (13.9%)
Craftswoman	531 (15.5%)	251 (18.2%)	782 (16.3%)
Employee	249 (7.3%)	147 (10.7%)	397 (8.3%)
Shopkeeper	194 (5.7%)	60 (4.4%)	254 (5.3%)
Housekeeper	615 (18.0%)	142 (10.3%)	757 (15.8%)
Housewife	1,174 (34.3%)	125 (9.0%)	1,299 (27.0%)
Middle/Upper class	55 (1.6%)	28 (2.0%)	84 (1.7%)
**Age of the mother, mean (sd)**	26.9 (4.9)	24.1 (4.7)	26.1 (5.0)
**Status, n (%)**			
Died before 31 y	1010 (29.5%)	633 (45.8%)	1643 (34.2%)
Died on [31; 99 y]	2324 (67.9%)	720 (52.1%)	3044 (63.4%)
Alive at 99 y	90 (2.6%)	28 (2.0%)	118 (2.5%)

¶: Paternal occupations for illegitimate births are imputed using MICE. Figures by occupation given in the Table are averaged over the ten completed datasets.

### Statistical analysis

#### Survival functions

The 4,805 individuals are included at birth, and fall into three categories: died before 31 y (with unknown age at death), died on [31; 99 y] (with known age at death), alive at 99 y. Any survival function S(t) is thus known at age 0 and on [31; 99 y]. This enables the computation of the median age at death in groups defined by legitimacy status, paternal occupation or maternal occupation. Bootstrap 95% confidence intervals (95% CI) were computed by first bootstrapping the data (with B = 500 bootstrap samples), and then performing multiple imputation on each bootstrap sample, as recommended by recent work [14].

#### Survival to 31 years

Probability of survival to age 31 y was modelled with a Generalized Additive Model (GAM) [15, 16]. A smooth function of the date of birth was included in the predictor to capture the effect of potential unmeasured time-varying explanatory variables [17]. The model was thus:

$$log\;\left(\frac{\pi_{31}}{{1-\pi }_{31}}\right)=PO+f_{1}(DB)+f_{2}(AM)+MO+Sex+Illeg+Arr$$ (1)

π_31_ is the probability to survive to age 31 y, *PO* the paternal occupation at the time of the birth, *DB* the date of birth (time since August 1^st^ 1914), *AM* the age of the mother at birth, *MO* the maternal occupation, *Illeg* a dummy variable taking value 1 if the birth is illegitimate, and *Arr* a normally distributed random effect controlling for the district of birth. Smooth functions *f*_*1*_ and *f*_*2*_ are represented as penalized cubic regression splines with respectively 100 and 8 degrees of freedom and evenly spaced knots. The smoothing parameters determining the effective degree of freedom of *f*_*1*_ and *f*_*2*_ are both selected to minimize the unbiased risk estimator score.

#### Survival after 31 years

The hazard ratio (HR) on the age span [31; 99 y] was regressed on explicatory variables using a mixed effects Cox proportional hazards model:

$$h(t)=h_{0}(t)\;exp(PO+DB+AM+MO+Sex+Illeg+Arr)$$ (2)

The notations are the same as above.

#### Differences in life expectancy at 31 years

To give adjusted estimates of life expectancy at 31 y (L_31_) by parental occupation, we fitted GAMs with Gaussian errors, identity link and the same predictors as in model (1), with smoothing parameters selected by generalized cross validation.

Because we had to impute all paternal occupations for illegitimate children, all analyses were performed on all children, and on legitimate children alone. Analysis of deviance was used to give a global assessment of the effect of paternal and maternal occupations. In order to test for sex-specific effects, models were also fitted separately for males and females. Finally, analyses of life expectancy at 31 y were performed on all those alive at 31 y (those who died on [31; 99 y] + those classified as alive at 99 y), and those died on [31; 99 y] only. Analyses were performed in R, using the following packages: *mice* for multiple imputation [11], *mgcv* for GAM regressions [18], and *coxme* for proportional hazard models [19]. All confidence intervals given are 95% confidence intervals.

## Results

### Characteristics of subjects

Table 1 provides the main characteristics of the studied individuals. Among legitimate children (for which both parental occupations are noted on the birth certificate), maternal and paternal occupations were correlated (Cramer’s V = 0.29; S1 Table). We found that 45.8% [43.2–48.5] of illegitimate children died before 31 y versus 29.5% [28.0–31.1] of legitimate children.

### Total survival experience by parental occupations and legitimacy status

**Fig 2:**
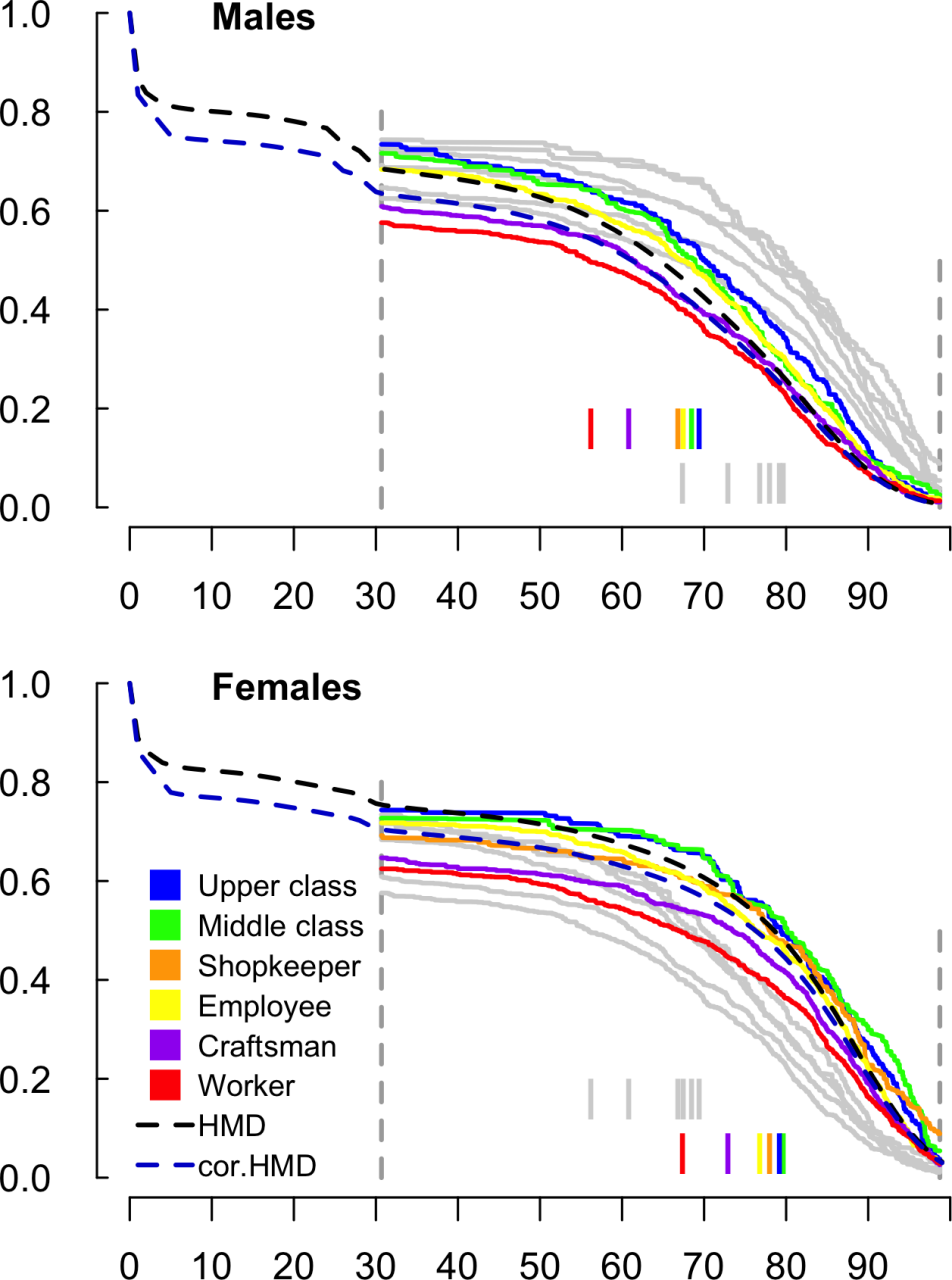
Survival curves by sex and occupation of the father. The survival functions are based on the pooled 10 completed datasets created by multiple imputation. In each plot, grey lines are survival functions for the other sex. Estimates of median ages at death based on B = 500 bootstrap samples are drawn in the lower part of the plots. The survival functions of the French 1915 male and female cohorts as given by the Human Mortality Database (HMD) are also drawn. Child mortality was known to be higher in the “departement” (district) of Paris than nationwide at the beginning of the XX^th^ century. Also drawn are HMD survival functions corrected for this excess child mortality (cor. HMD), based on the quotients of mortality _1_q_0_ and _4_q_1_ as given by ref. 20, which relate to the 1901–1905 female cohort. 1915 HMD _1_q_0_ and _4_q_1_ were corrected assuming that the percent excess Parisian mortality for both sexes was the same in 1915 as in 1901–1905.

Survival varied strongly with paternal occupation (Fig 2). The highest median age at death (MAD) among women was 79.7 y [72.9–84.5], attained by those born to middle class fathers. The highest MAD among men was 69.4 y [64.9–73.8] for those born to upper class fathers. In both sexes, MAD was the lowest when the father was a worker (Men: 56.3 y [48.6–62.7]; women: 67.4 y [60.8–72.7]) (Fig 2).

MAD of illegitimate children was 18.9 y [13.3–32.3] years below that of legitimate children (illegitimate: 53.2 y [47.9–66.6]; legitimate: 72.1 y [71.0–73.4]). MAD also varied according to maternal occupation in both sexes (S1 Fig).

### Survival to 31 years

Survival to 31 y was significantly associated with paternal occupation in all 10 datasets completed by multiple imputation (analysis of deviance: median of the 10 p-values (p) = 7.8 x 10^−6^; maximum p = 5.3 x 10^−4^). This was largely due to low survival in worker’s offspring: indeed, the pooled adjusted odds ratio (OR, with craftsmen as the group of reference) was 0.77 [0.63–0.94] for those born to a worker. Though not significant, there was a trend for higher survival for those born to a middle class father (OR = 1.33 [0.89–2.00]) and upper class father (OR = 1.23 [0.87–1.74]). The offspring of craftsmen, employees and shopkeepers experienced intermediate survival (Table 2 and S2 Fig). Consistent with the idea that association between paternal occupation and survival to 31 y was largely driven by the high mortality in workers’ offspring, the analysis of deviance was not significant when they were excluded from the dataset (median p = 0.14).

**Table 2:** Parametric terms from model (1) and model (2).

	OR of survival to 31 y (95% CI)	HR on [31; 99 y] (95% CI)
**Fatherly occupation**		
Worker	0.77 (0.63–0.94)	1.06 (0.94–1.19)
Craftsman	1 (ref)	1 (ref)
Employee	1.18 (0.94–1.46)	1.01 (0.89–1.14)
Shopkeeper	1.06 (0.81–1.38)	0.83 (0.69–1.00)
Middle class	1.33 (0.89–2.00)	0.87 (0.72–1.06)
Upper class	1.23 (0.87–1.74)	0.94 (0.78–1.12)
**Motherly occupation**		
Worker	0.82 (0.65–1.04)	1.07 (0.93–1.24)
Servant	0.85 (0.68–1.07)	0.98 (0.86–1.13)
Craftswoman	1 (ref)	1 (ref)
Employee	0.96 (0.73–1.25)	0.89 (0.76–1.04)
Shopkeeper	1.04 (0.75–1.45)	1.16 (0.95–1.42)
Housekeeper	0.91 (0.73–1.13)	1.01 (0.89–1.15)
Housewife	1.05 (0.84–1.31)	0.97 (0.86–1.09)
Middle & upper class	1.32 (0.77–2.29)	0.94 (0.71–1.24)
**Sex**		
M	0.84 (0.74–0.95)	1.63 (1.52–1.76)
F	1 (ref)	1 (ref)
**Legitimacy**		
Legitimate	1 (ref)	1 (ref)
Illegitimate	0.54 (0.46–0.62)	1.06 (0.96–1.16)

Probability of survival to age 31 y (model 1) and the hazard ratio on [31; 99 y] (model 2) were modeled with Generalized Additive Models on each of the 10 datasets completed by multiple imputation. Adjusted odds ratios (OR) and hazard ratios (HR) obtained on each dataset were then combined by Rubin’s rules.

Survival to age 31 y was also strongly decreased in illegitimate children: the adjusted OR with legitimate children was 0.54 [0.46–0.62] (Table 2).

Maternal occupation had no effect (median p = 0.31, minimum p = 0.14). The age of the mother was found to be linearly associated with survival in all datasets (effective degree of freedom of f_2_ after penalization ≈1). There was a 1.09 [1.02–1.16] fold increase in survival associated with each 5-year increase in the age of the mother.

The results remained unchanged when the analysis was restricted to legitimate births alone. In particular, paternal occupation was still found associated with survival (analysis of deviance: p = 1.3 x 10^−4^), again due to low survival in worker’s offspring (S2 Fig).

### Survival after 31 years

**Fig 3:**
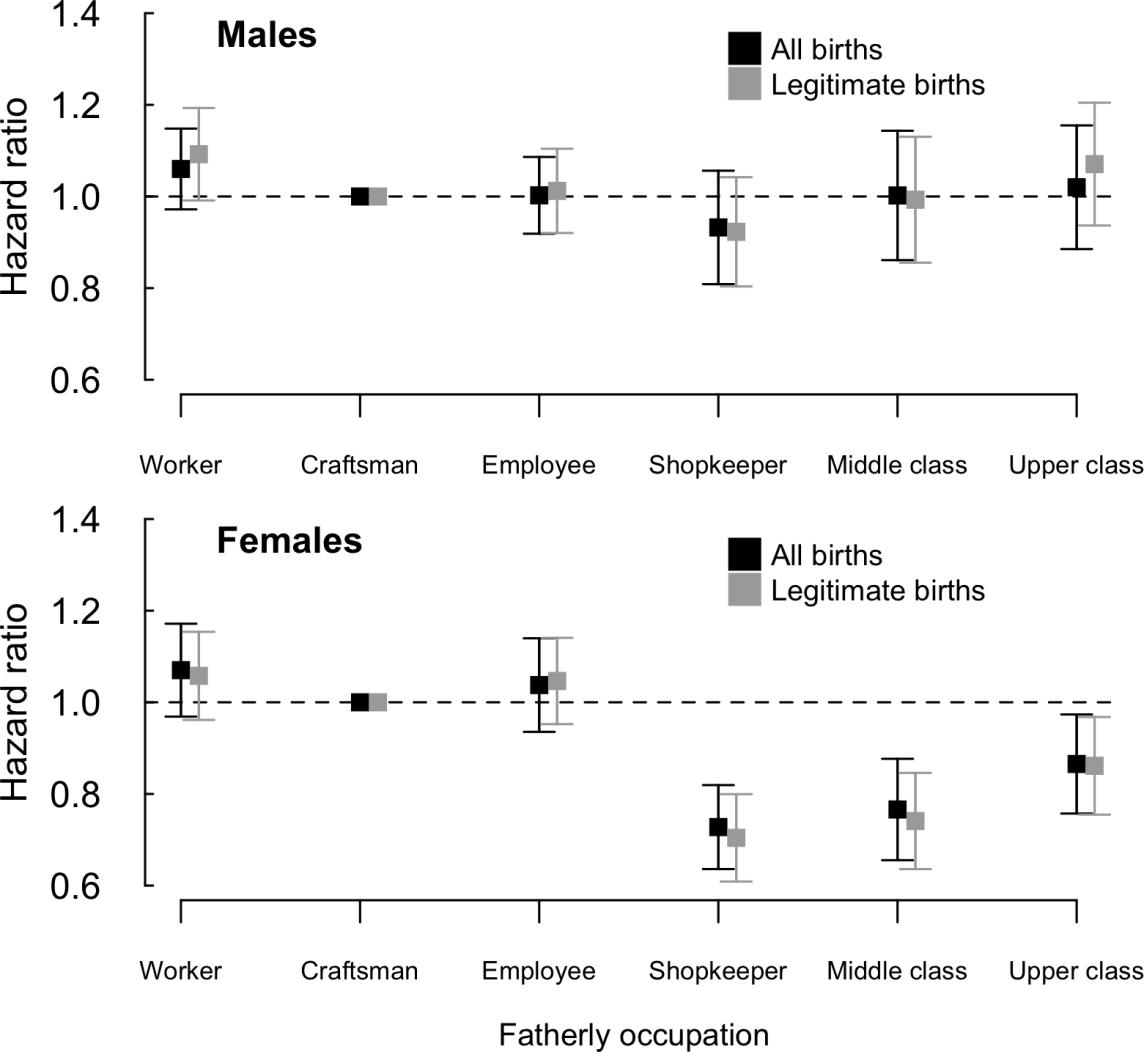
Adjusted hazard ratio according to sex and paternal occupation. The effect of paternal occupation on the hazard ratio on [31; 99 y] is estimated for each completed dataset thanks to a Cox proportional hazard model, and then combined following Rubin’s rules. Also given are estimates on legitimate children alone, for whom no imputation of paternal occupations is needed. For both sexes, the reference group is that of craftsmen’ children. Estimated effects are given ± standard error.

The hazard ratio (HR) was associated with paternal occupation (median p = 9.0 x 10^−3^; maximum p = 7.8 x 10^−2^). In the by-sex analysis, the effect of paternal occupation was found to be significant for females (median p = 5.2 x 10^−4^), not for males (median p = 0.73). As shown on Fig 3, the HR among males was very close in all groups. In contrast, a clear difference was visible among women between the three lower status groups (worker, employee, craftsman) and the three highest (shopkeeper, middle and upper class). The life expectancy analysis was consistent with these results (S3 Fig).

The results held true when the analysis was restricted to legitimate births alone. In particular, an association was found between paternal occupation and survival after age 31 y in females (p = 3.2 x 10^−3^), but not in males (median p = 0.74; see Fig 3).

## Discussion

This study reports that parental occupations and legitimacy are strongly associated with whole life mortality for a cohort born a century ago in Paris. The association was observed for mortality after age 31 y as well as before age 31 y.

Because birth registers do not indicate the date of death when it occurred before 1945, the probability of survival to 31 y was our indicator of mortality from birth to young adulthood. Unskilled paternal occupation (‘workers’ category, which forms the lowest quarter of paternal occupations) and illegitimacy were independently associated with a strong excess risk of death before 31 y. We observed no effect of maternal occupation on survival to 31 y after adjustment for paternal occupation. It is likely that most of the differences in survival to 31 y according to paternal occupation and legitimacy relate to early life mortality, since the main contributors to the probability of death before 31 y in our study were infant (0–1 y) and child (1–5 y) mortalities, and to a lesser extent World War II (WW2) (see Fig 2, HMD survival curves).

In the context of 1914 Paris, both paternal occupation and illegitimacy were likely to influence many environmental factors, including pre and postnatal nutrition, risk of infections (in particular during the 1918 influenza pandemic) and access to healthcare. About 20% of infants were left to wet-nurses mainly residing outside Paris [20]. Illegitimacy increased the probability of being sent to a wet-nurse [21]. Quality of wet-nursing was known to depend directly on the household income [22], and in turn was critical to child’s health. Risk of diarrheas (~ 25% of infant deaths nationwide [23]) was increased for bottle-fed infants if sterilization was not performed by the parents or wet-nurse. It may also be the case that the father’s environmental exposures affected the offspring’s biological vulnerability in early life through transgenerational epigenetic modifications [24–26].

Lack of effect of maternal occupation on survival to 31 y was unexpected. Our interpretation is that it is due to the fact that 43% of mothers are recorded on birth certificates as “housekeeper” or “housewife”, a status that may indeed mask heterogeneous socioeconomic conditions and wealth. This is evidenced by the diversity of paternal occupations associated with a “housekeeper” or a “housewife” (see S1 Table). Interestingly, survival was not improved when the mother was a “housekeeper” or a “housewife” compared with employed mothers although they were more available for direct childcare (notably breastfeeding). It may also be noted that the offspring of workers, among whom were women working in war factories, had a survival to 31 y close to that of servants’ offspring, a fact that supports the idea that work in a war factory during pregnancy was not a key factor in infant mortality, contrary to what was held by some leading pediatricians of the time [27]. The association found between low maternal age and early life mortality is still observed in contemporary populations [28], despite changes in the distribution of maternal ages, and has been attributed to decreased maternal care [29] and biological immaturity resulting in low birth weight [30].

For those people who survived WW2, knowledge of the exact date of death after 1945 allowed classical survival analysis through two different approaches to study mortality after 31 y (modeling the hazard ratio on [31; 99 y] and modeling the life expectancy at 31 y). Both approaches showed an association between paternal occupation at birth and mortality for females, but not for males. Neither legitimacy nor maternal occupation showed an association with mortality after 31 y.

Loose classification or loose imputation of paternal occupations cannot account for this absence of differences among men after 31 y, given that we do find differences according to paternal occupation for both sexes before 31 y, and among women after 31 y. Moreover, results were not changed when the analysis was restricted to legitimate children alone. One hypothesis is that the men of the cohort encountered in adulthood risk factors that were strong enough to overcome the effects of paternal SES. Indeed, smoking and stress are likely to have been stronger mortality “equalizers” among males than among females. In 1953, 72% of French men (only 17% of women) were regular smokers [31]. On the other side, most females born 1914–1916 into middle and upper class families never entered the workforce [23], thereby remaining protected from the physical and psychosocial stress associated with employed work, contrary to women of the working class.

Although the current study allows no identification of its causes, we wondered if the sexual dimorphism observed for mortality after 31 y could relate in part to the wartime conditions at the time of the birth. Most young women had their husband called to arms in the early months of the conflict. These women had to face loneliness during pregnancy, fear for husband’s death and the day-to-day challenges of single life, exposing their offspring to socially dependent degree of early life adversity and childhood stress. Altered perinatal maternal behavior is known to modify the offspring’s stress response durably [32, 33], and to yield increased susceptibility to diseases in adulthood [34–36]. Some consequences of prenatal maternal stress have been shown to vary according to sex on animal models [37–39], and occasionally in humans [40]. Disentangling their potential contribution to that of the aforementioned conditions lived later in life could be achieved by studying individuals born shortly before the War, e.g. around 1910.

In conclusion, the observation of this cohort over its entire lifetime found large differences in median ages at death between children of both sexes born to parents of high and low socioeconomic status. Most of these differences were due to mortality occurring during youth, likely to be due to direct environmental accidents hitting more vulnerable offspring of both sexes. Differential mortality was also observed after 31 y for women only, an unexpected observation that calls for further studies of extinct cohorts in other historical contexts. While our results show that social inequalities in the early 20^th^ century translated in decreased longevity in offspring of those unprivileged, nobody can predict how these findings can be extrapolated to children born in contemporary countries. Some of the signals generated by the lowest SES situations may still act on infants in contemporary societies through biological, educational, or behavioral pathways comparable to that which prevailed for those born in 1914 France. In contrast, newly appeared conditions that have today strong links with SES, such as obesity [41], may affect offspring's longevity through socially dependent mechanisms that were not prevalent a century ago. Causal mechanisms for such long-term differences in longevity may involve the developmental effects of socially dependent environmental cues on phenotypic plasticity.

## Supporting information

S1 Fig Median age at death according to maternal occupation and sex. Median ages at death are computed separately for males and females according to maternal occupation at the time of birth. For both sexes, those born of a middle & upper class mother have the highest median age at death. Dotted line: Males = Females.
(PDF)

S2 Fig Survival to 31 y by paternal occupation (all births / legitimate births). Probability of survival to 31 y was modeled with a Generalized Additive Model (GAM) on each of the 10 datasets completed by multiple imputation. The model was fitted for all births and for legitimate births only (which do not necessitate imputation of paternal occupations). Association of survival to 31 y with paternal occupation was not changed when the analysis was restricted to legitimate births alone. Plotted standard errors are those for the all births estimates and are computed thanks to the delta method.
(TIFF)

S3 Fig Life expectancy at 31 y by paternal occupation. The life expectancy at 31 y was modeled with a Generalized Additive Model separately for each sex on each completed dataset and then combined. Since March 29^th^ 1945, dates of deaths have been notified on birth certificates. [31; 99 y] is the age range on which the deaths of all the members of the cohort were observed. Those with no date of death on their birth certificate may have died before March 29^th^ 1945 or be alive at the end of the observation period. Those with no life event (marriage, divorce, guardianship) after March 29^th^ 1945 were considered dead before March 29^th^ 1945. Conversely, those with no date of death but at least one life event after March 29^th^ 1945 were considered alive at age 99 y. To test the effect of this indirect means of classification, the analysis of life expectancy at 31 y was performed on all those considered alive at 31 y (black) and was then restricted to those who died on [31; 99 y] (grey), with hardly any difference in the results. These results are in line with those obtained from the modeling of the hazard ratio on the age span [31; 99 y]: increased variability according to paternal occupation is found among females. Estimated effects are given ± standard error.
(TIFF)

S1 File Dataset used. (CSV)

S1 Table Contingency table for parental occupations of legitimate children. Both paternal and maternal occupations are available on the birth certificate of virtually all legitimate children. 0.93% of all legitimate children (N = 32) with at least one missing or unclassified parental occupation were excluded from the contingency table. Cramer’s V is 0.29.
(DOCX)
