# Supplementary figures and images for: Impact of social inequalities at birth on the longevity of children born 1914–1916: A cohort study
Source: PLoS One. 2017 Oct 16;12(10):e0185848. doi: 10.1371/journal.pone.0185848 (PMC5643053; doi:10.1371/journal.pone.0185848)

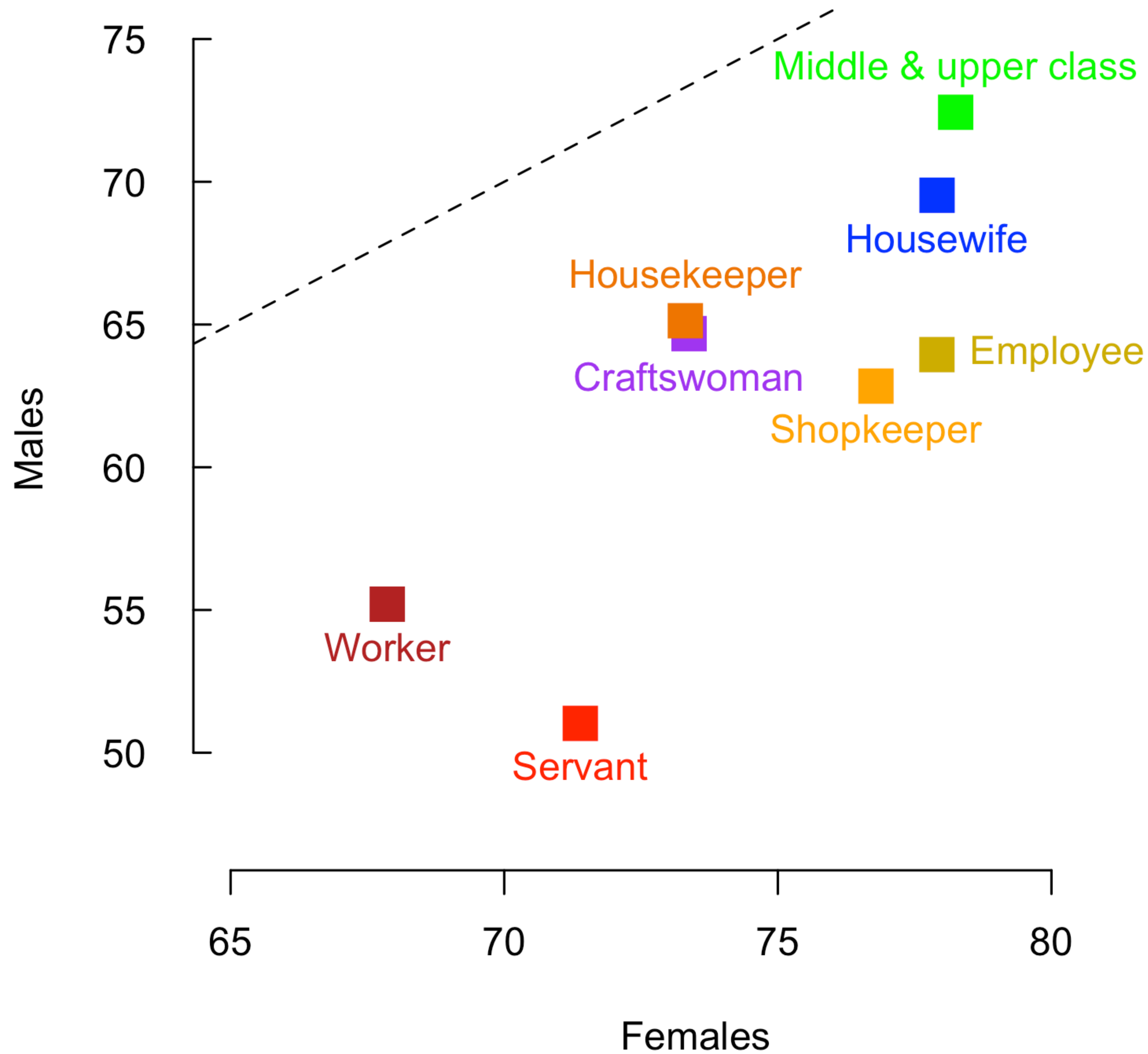

Supplement: S1 Fig — Median ages at death are computed separately for males and females according to maternal occupation at the time of birth. For both sexes, those born of a middle & upper class mother have the highest median age at death. Dotted line: Males = Females. (PDF) [file pone.0185848.s001.pdf]

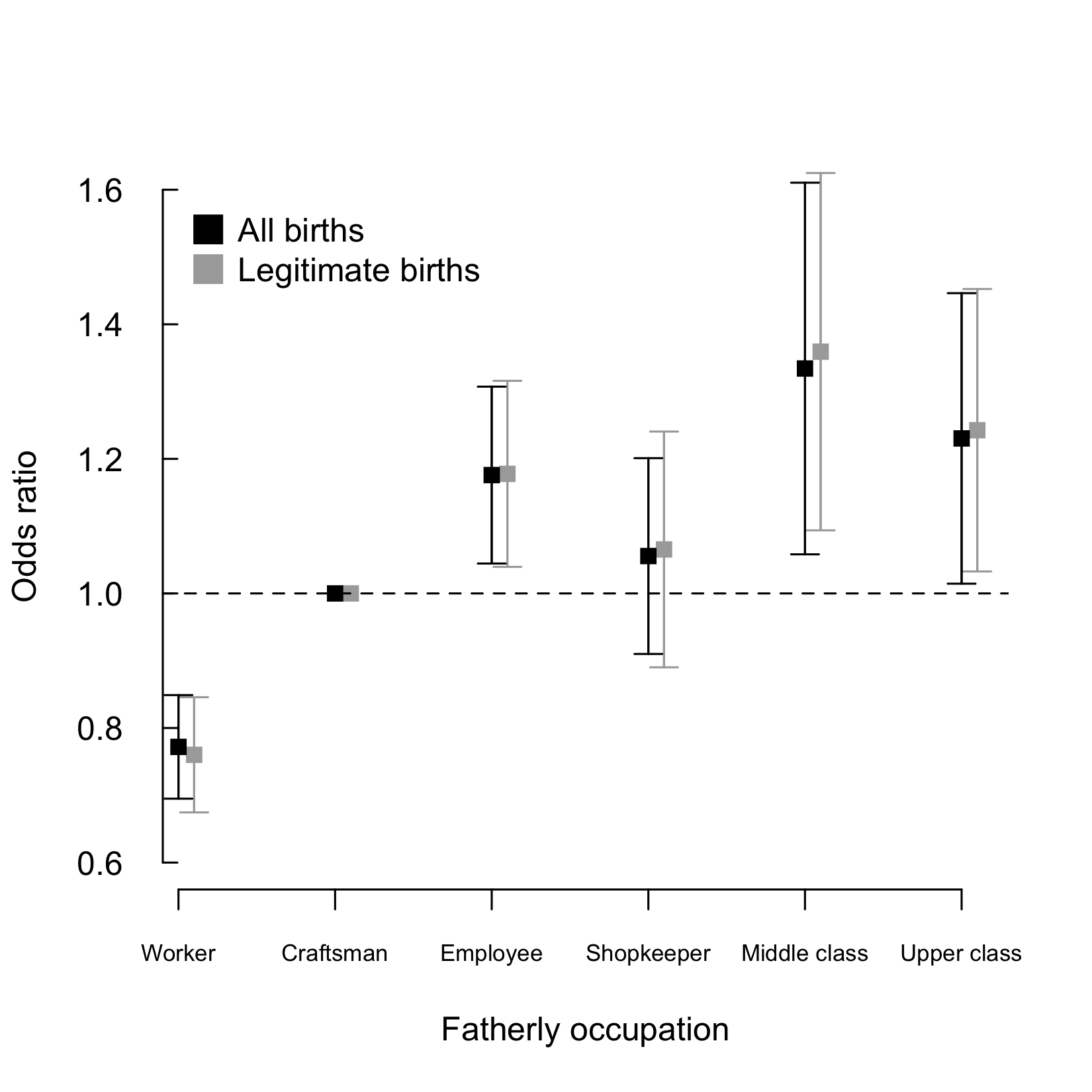

Supplement: S2 Fig — Probability of survival to 31 y was modeled with a Generalized Additive Model (GAM) on each of the 10 datasets completed by multiple imputation. The model was fitted for all births and for legitimate births only (which do not necessitate imputation of paternal occupations). Association of survival to 31 y with paternal occupation was not changed when the analysis was restricted to legitimate births alone. Plotted standard errors are those for the all births estimates and are computed thanks to the delta method. (TIFF) [file pone.0185848.s002.tiff]

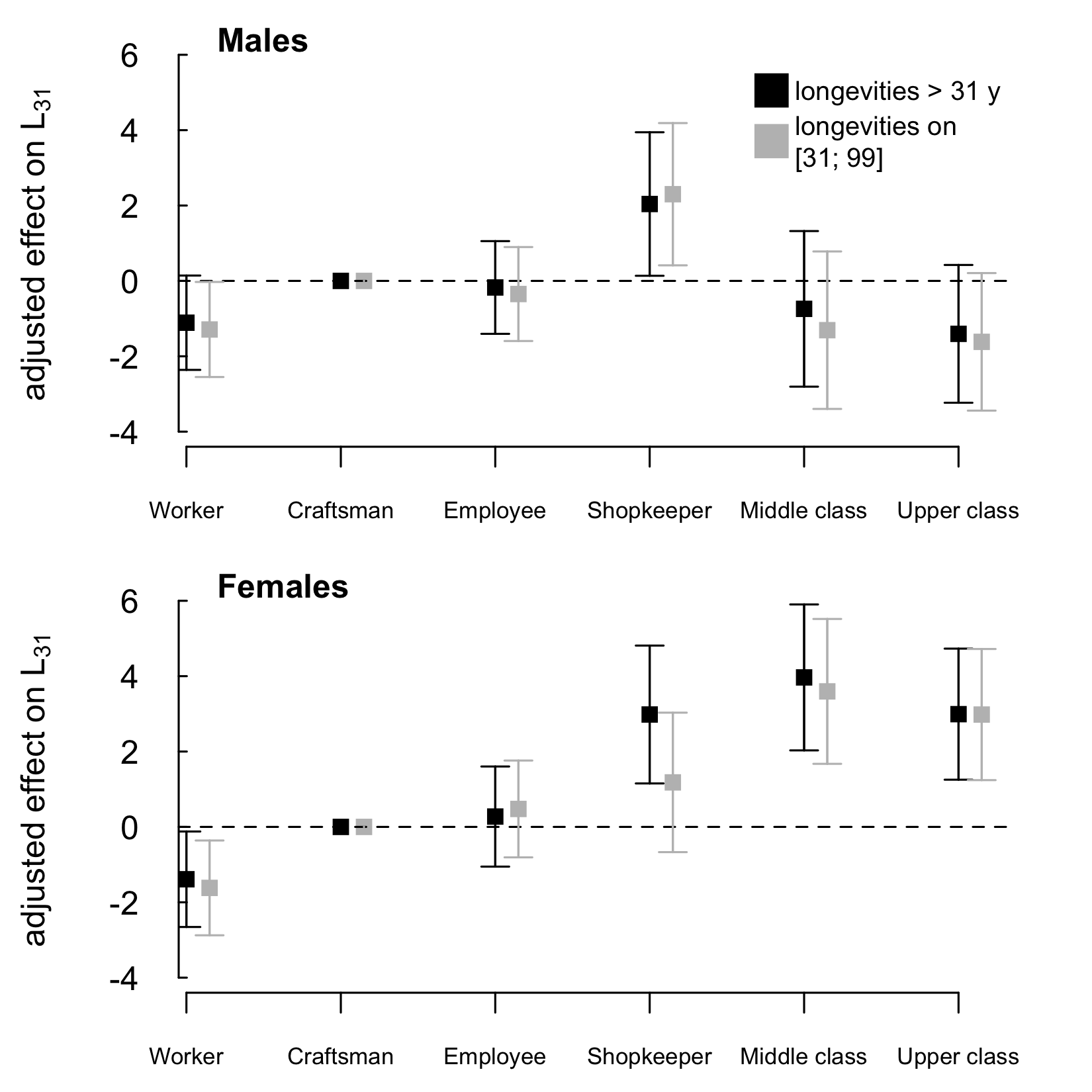

Supplement: S3 Fig — The life expectancy at 31 y was modeled with a Generalized Additive Model separately for each sex on each completed dataset and then combined. Since March 29th 1945, dates of deaths have been notified on birth certificates. [31; 99 y] is the age range on which the deaths of all the members of the cohort were observed. Those with no date of death on their birth certificate may have died before March 29th 1945 or be alive at the end of the observation period. Those with no life event (marriage, divorce, guardianship) after March 29th 1945 were considered dead before March 29th 1945. Conversely, those with no date of death but at least one life event after March 29th 1945 were considered alive at age 99 y. To test the effect of this indirect means of classification, the analysis of life expectancy at 31 y was performed on all those considered alive at 31 y (black) and was then restricted to those who died on [31; 99 y] (grey), with hardly any difference in the results. These results are in line with those obtained from the modeling of the hazard ratio on the age span [31; 99 y]: increased variability according to paternal occupation is found among females. Estimated effects are given ± standard error. (TIFF) [file pone.0185848.s003.tiff]
