# Supplementary material for: Impact of social inequalities at birth on the longevity of children born 1914–1916: A cohort study
Source: PLoS One. 2017 Oct 16;12(10):e0185848. doi: 10.1371/journal.pone.0185848 (PMC5643053; doi:10.1371/journal.pone.0185848)
Supplement: S1 Table — Both paternal and maternal occupations are available on the birth certificate of virtually all legitimate children. 0.93% of all legitimate children (N = 32) with at least one missing or unclassified parental occupation were excluded from the contingency table. Cramer’s V is 0.29. (DOCX) [file pone.0185848.s005.docx]

**S1 Table. Contingency table for parental occupations of legitimate children**

| **Father**  **Mother** | Worker | Craftsman | Employee | Shopkeeper | Middle Class | Upper Class |
| --- | --- | --- | --- | --- | --- | --- |
| Worker | 199 (20.2% ¶) | 84 (14.3%) | 54 (5.5%) | 16 (5.1%) | 8 (4.1%) | 1 (0.3%) |
| Servant | 76 (7.7%) | 26 (4.4%) | 119 (12.2%) | 14 (4.5%) | 4 (2.0%) | 0 |
| Craftswoman | 159 (16.2%) | 148 (25.2%) | 157 (16.1%) | 30 (9.6%) | 30 (15.2%) | 2 (0.6%) |
| Employee | 45 (4.6%) | 39 (6.6%) | 118 (12.1%) | 25 (8.0%) | 15 (7.6%) | 5 (1.5%) |
| Shopkeeper | 37 (3.8%) | 20 (3.4%) | 26 (2.7%) | 102 (32.8%) | 5 (2.5%) | 3 (0.9%) |
| Housekeeper | 259 (26.3%) | 116 (19.7%) | 175 (17.9%) | 39 (12.5%) | 17 (8.6%) | 3 (0.9%) |
| Housewife | 206 (21.0%) | 148 (25.2%) | 309 (31.7%) | 85 (27.3%) | 106 (53.8%) | 307 (90.8%) |
| Middle &  Upper class | 2 (0.2%) | 7 (1.2%) | 17 (1.7%) | 0 (0.0%) | 12 (6.1%) | 17 (5.0%) |

¶ Frequencies in the Table are for paternal occupation alone (within a column).
